# Love is in the hair: arginine methylation of human hair proteins as novel cardiovascular biomarkers

**DOI:** 10.1007/s00726-021-03024-5

**Published:** 2021-06-28

**Authors:** Alistair James Marsden, David R. J. Riley, Stefan Birkett, Quentin Rodriguez-Barucg, Barbara-ann Guinn, Sean Carroll, Lee Ingle, Thozhukat Sathyapalan, Pedro Beltran-Alvarez

**Affiliations:** 1 Department of Biomedical Sciences, University of Hull, Cottingham Rd, Hull, HU6 7RX UK; 2 Department of Sport, Health and Exercise Science, University of Hull, Hull, UK; 3 School of Sport and Health Sciences, University of Central Lancashire, Preston, UK; 4 Academic Endocrinology, Diabetes and Metabolism, Hull York Medical School, University of Hull, Hull, UK

**Keywords:** Arginine methylation (ArgMe), Asymmetric dimethylarginine (ADMA), Biomarker, Cardiovascular disease (CVD), Hair, Post-translational modifications (PTM), Protein

## Abstract

Cardiovascular disease is the major cause of death worldwide. Extensive cardiovascular biomarkers are available using blood tests but very few, if any, investigations have described non-invasive tests for cardiovascular biomarkers based on readily available hair samples. Here we show, first, that human hair proteins are post-translationally modified by arginine methylation (ArgMe). Using western blot, proteomic data mining and mass spectrometry, we identify several ArgMe events in hair proteins and we show that keratin-83 is extensively modified by ArgMe in the human hair. Second, using a preliminary cohort (*n* = 18) of heterogenous healthy donors, we show that the levels of protein ArgMe in hair correlate with serum concentrations of a well-established cardiovascular biomarker, asymmetric dimethylarginine (ADMA). Compared to blood collection, hair sampling is cheaper, simpler, requires minimal training and carries less health and safety and ethical risks. For these reasons, developing the potential of hair protein ArgMe as clinically useful cardiovascular biomarkers through further research could be useful in future prevention and diagnosis of cardiovascular disease.

**Supplementary Information:**

The online version contains supplementary material available at 10.1007/s00726-021-03024-5.

## Introduction

Cardiovascular disease (CVD) is still the leading cause of death in industrialised countries and is associated with enormous public health and economic costs (Virani et al. 2020). Globally, the proportion of deaths due to CVD is growing (Roth et al. 2019). Many biomarkers of cardiovascular health and disease have been developed, including B-type natriuretic peptide, markers of oxidative stress, inflammatory mediators and metabolic biomarkers (Hirata et al. 2020), among others (Adamcova and Šimko 2018). These measurements are almost always done on serum and therefore require invasive procedures and specialised personnel, with variable assay costs.

In contrast, over the past few years, hair monitoring has gained visibility as a possible testing substrate for many diagnostic and forensic applications (Adeola et al. 2018). Hair collection is simple, non-invasive and can be performed by minimally trained personnel. Hair samples can be stored long-term without the need for laboratory space or equipment and, compared to blood samples, carry no, or very little, health and safety risks for the experimenter and/or the environment.

Hair consists mainly of proteins (Chen and Yu 2020). Protein post-translational modifications (PTMs) are critical players in protein biochemistry, can change protein structure, activity, localization and interactions (Onwuli and Beltran-Alvarez, 2016), and can modify proteins in many tissues, including hair (Adav et al. 2018). In our laboratory, we are studying a particular PTM known as arginine methylation (ArgMe). Protein ArgMe consists of the transfer of a methyl group (CH_3_) from S-adenosyl-l-methionine to the side chain guanidino nitrogen of arginine, in an enzymatic reaction catalysed by protein arginine methyltransferases (PRMTs), (Samuel et al. 2021). The addition of a CH_3_ group removes a potential hydrogen bond donor from the recipient arginine and produces bulkier and more hydrophobic methylarginine residues. In humans, there are three types of PRMTs that catalyse ArgMe, each responsible for a different ArgMe end-product. Type I and II PRMTs catalyse Arg monomethylation followed by, respectively, asymmetric and symmetric dimethylation. Type III PRMTs are defined as producing monomethylarginine residues only (Jarrold and Davies 2019). Type I PRMTs include PRMT1, -2, -3, -4, -6 and -8. PRMT5 and -9 are type II PRMTs and PRMT7 is accepted as the only type III PRMT (Zurita-Lopez et al. 2012).

The degradation of proteins modified by ArgMe leads to the proteolytic products N^G^-monomethyl-l-arginine (MMA), N^G^,N^G^-dimethyl-l-arginine (asymmetric dimethylarginine, ADMA) and N^G^,N′^G^-dimethyl-l-arginine (symmetric dimethylarginine, SDMA), and the levels of these circulating methylarginine metabolites are thought to correlate with the cellular concentration of proteins modified by ArgMe (Bollenbach et al. 2019; Post et al. 2021). These metabolites exercise a range of functions when released into the circulation, notably related to nitric oxide synthase inhibition. As such, high levels of methylarginine metabolites, such as ADMA, in serum are associated with endothelial dysfunction, provide well-known prognostic markers for cardiovascular risk and mortality (Jarzebska et al. 2019; Mangoni et al. 2021; Tsikas 2020) and correlate with cardiorespiratory dysfunction (Böger et al. 1997; Krempl et al. 2005; Leong et al. 2008; Meinitzer et al. 2007; Surdacki et al. 1999; Tajti et al. 2018; Yoo and Lee 2001; Zinellu et al. 2018).

To the best of our knowledge, ArgMe of hair proteins has not previously been reported. Here, we hypothesised that hair proteins are modified by ArgMe and we aimed to investigate any correlation between hair protein ArgMe and serum ADMA. This may provide a cardiovascular marker based on hair sampling, which provides a longer time of reference and is less susceptible to short-time diurnal or biological variability than blood or urine sampling. Given the impending perfect storm of climate change and a predicted increase in CVD over the next few decades (Watts et al. 2021), the identification of valid and reliable cardiovascular biomarkers in hair samples has the potential to enhance primary care, early diagnosis, prevention of disease development and management of CVD.

## Materials and methods

### Hair samples

Hair samples from healthy volunteers were collected from the posterior side of their head and as close as possible to the scalp within the context of University of Hull Faculty of Health Sciences ethically approved project, reference FHS57 and a Hull York Medical School ethically approved clinical study, reference 17_11. Inclusion criteria for the subjects were age (18–65 years), non-clinically obese BMI (between 19 and 29 kg/m^2^), and completion of informed consent. An additional inclusion criterium was length of hair at the posterior side of the scalp (> 5 cm), this is the likely reason why all participants were female. Exclusion criteria were subjects who had bleached or dyed their hair in the 6 months prior to sample collection, concurrent disease, alcohol intake within 24 h of donation and pregnancy. Approximately 40 individual hairs were collected from each participant, alongside 2–5 mL blood through venepuncture. Blood was incubated at room temperature for 30 min in upright tubes to allow for blood clotting, and serum was then collected by centrifugation at 16,000* g* for 20 min. Serum and hair samples were stored at -20 °C until being analysed.

### Protein lysis and western blot for hair protein ArgMe measurements

The closest centimetres to scalp of hair strands were manually minced into small (approximately 1 mm) fragments and proteins from at least 5 mg hair were extracted into 300 µl of buffer according to the Minute protein extraction kit for hair and nails (Invent Biotechnologies), which includes incubation in 0.5% SDS and 10% β-mercaptoethanol at 55 °C for > 24 h. Protein lysates were separated through 10–12% SDS-PAGE along a protein marker (#26619, Thermofisher) and proteins transferred to PVDF membranes (GE Healthcare). We used two validated α-ArgMe antibodies (#8015 and #8711, Cell Signalling Technologies) and an α-keratin-83 antibody (ab174272, Abcam) at 1:1000 dilutions in 5% non-fat milk. Quantification of protein band intensity and the overlap of ArgMe and keratin-83 intensities were done using ImageJ. All samples were electrophoresed down a parallel gel, which was silver-stained for total protein normalisation. Inter-gel quantifications were done with the aid of internal controls.

### ELISA for ADMA measurements

ADMA levels were measured in serum, in duplicate, using a commercial ELISA kit (Abcam, #ab213972). Synthetic ADMA standards for calibration were purchased from Merck. Raw calibration measurements and a four-parameter logistic curve fitting are shown in Supp. Figure 1.

### MaxQuant analysis of protein ArgMe

The bioinformatic analysis was completed following published protocols (Onwuli et al. 2017). Briefly, proteomic raw data from PXD007224 (Adav et al. 2018) and PXD016169 (Plott et al. 2020) were downloaded from the PRIDE proteomics database and mined for protein ArgMe using MaxQuant (v1.6.14.0 and v1.6.17.0 for PXD007224 and PXD016169, respectively). ArgMe was set as a variable modification together with Met oxidation and N-terminal protein acetylation. Cys carbamidomethylation was set as a fixed modification. MaxQuant parameters were set as default in qualitative searches against the human proteome (GRCh38 and UP000005640, 20610 proteins, March 2021 for PXD007224 and PXD016169, respectively).

### Mass spectrometry analysis of keratin-83

Hair lysates were resolved by SDS-PAGE and the gel stained using GelCode blue stain reagent (Thermo). The band putatively corresponding to keratin-83 at around 60 kDa was excised and destained. Proteins therein were reduced, alkylated and trypsinised (Pierce) overnight at 37 °C. Peptides were desalted and loaded onto a 50 cm EasyNano PepMap column driven by a Water mClass UPLC, with elution over 1 h onto an Orbitrap Fusion Tribrid mass spectrometer operated in data-dependent acquisition (DDA) mode. Resulting spectra were searched using Mascot against the human subset of the UniProt database. Oxidation of Met and mono- and dimethylation of Arg were specified as variable modifications. Trypsin specificity was set as semi- and missed cleavages were set to a maximum of four. Scaffold PTM (version 5.0.1, Proteome Software) was then used to annotate PTM sites derived from MS/MS sequencing results.

## Results

### Hair proteins are modified by ArgMe

To gain an understanding of the scope of protein ArgMe in human hair, we searched proteomics datasets of the hair proteome from healthy adult individuals (PXD007224 and PXD016169 from Adav et al. (2018) and Plott et al. (2020), respectively) for ArgMe. Using our recently published proteomics workflow (Onwuli et al., 2017), we identified tens of ArgMe sites in hair proteins (Supp. Tables 1 and 2). We counted 4 ArgMe sites in 4 proteins that were found in both the PXD007224 and the PXD016169 datasets (Table 1), including on keratin-83, one of the most abundant proteins in hair (Moll et al. 2008).

**Table 1:** ArgMe sites identified in both PXD007224 and PXD016169

Gene name	Protein name	Molecular weight (kDa)	Mono-ArgMe sites	Di-ArgMe sites
KRT33A	Keratin, type I cuticular Ha3-I	45.9	173	Not in common
KRT86	Keratin, type II cuticular Hb6	53.5	21	Not in common
KRT83	Keratin, type II cuticular Hb3	54.2	26	Not in common
HDAC5	Histone deacetylase 5	122.0	Not in common	151

**Fig. 1:**
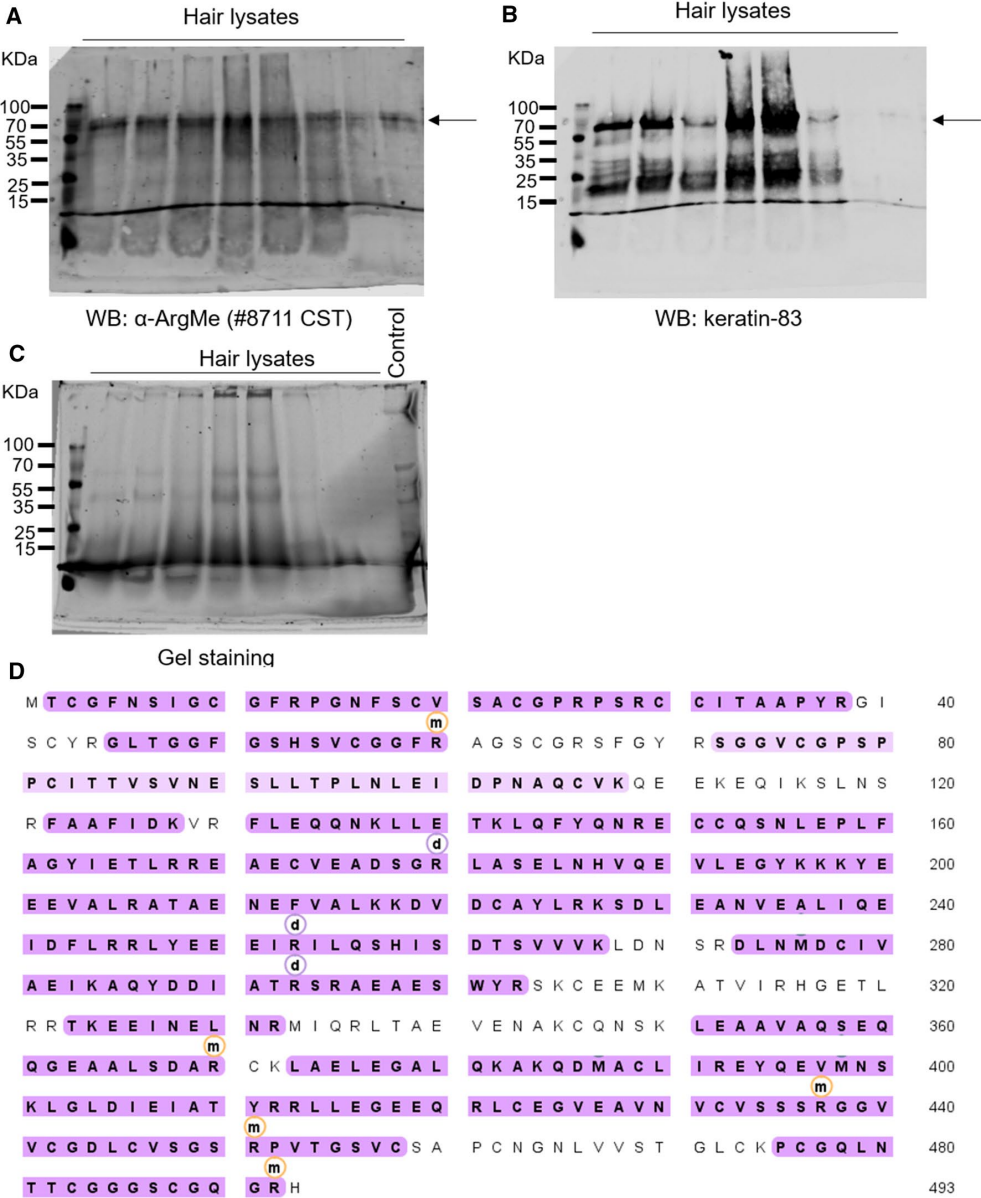
**A** Western blot showing detection of hair protein ArgMe from 8 healthy volunteers. An antibody targeting protein ArgMe recognised a major protein band between the 55 and 70 kDa markers (indicated by the arrow). **B** Same participants and same membrane as in (**A**) blotted using an antibody targeting keratin-83, a universal hair protein of expected MW of 54.2 KDa and detected between the 55 and 70 kDa markers (arrow). See Supp. Figure 2 for a higher exposure and Supp. Figure 3 for the overlap of the ArgMe and the keratin-83 panels. **C**. Silver staining of a gel loaded with the same samples as in (**A**) and (**B**). Twenty µg of proteins were loaded in the control lane for comparison with hair protein loading. **D**. Sequence coverage of keratin-83 mass spectrometry analysis (shaded residues, 81% of all keratin-83 sequence). Arginine residues found to be modified by mono- and di-ArgMe are identified with *m* and *d* labels, respectively, and include: R60, R180, R253, R293, R370, R437, R451 and R492

To provide direct evidence that hair proteins are modified by ArgMe, we collected hair samples from healthy participants and searched for hair protein ArgMe using western blotting. We routinely observed several protein bands in the range of 55–70 kDa recognised by antibodies specific for mono-ArgMe (Fig. 1).

We hypothesised that one of the major bands recognised by the α-ArgMe antibodies may correspond to keratin-83 and an antibody against keratin-83 appeared to recognise the same protein band as the α-ArgMe antibody at 55–70 kDa (Fig. 1 and Supp. Figures 2 and 3). The keratin-83 antibody recognised other bands below 35 kDa, which may correspond to protein degradation under the harsh conditions used for protein extraction from hair (Navone and Speight 2018). We then isolated keratin-83 from human hair lysates and analysed the protein by mass spectrometry. We found that keratin-83 was extensively modified by ArgMe (Fig. 1D, Supp. Figure 4 and Supp. Table 3). This is the first report of ArgMe in hair proteins.

### Levels of hair protein ArgMe correlate with serum ADMA concentrations

**Fig. 2:**
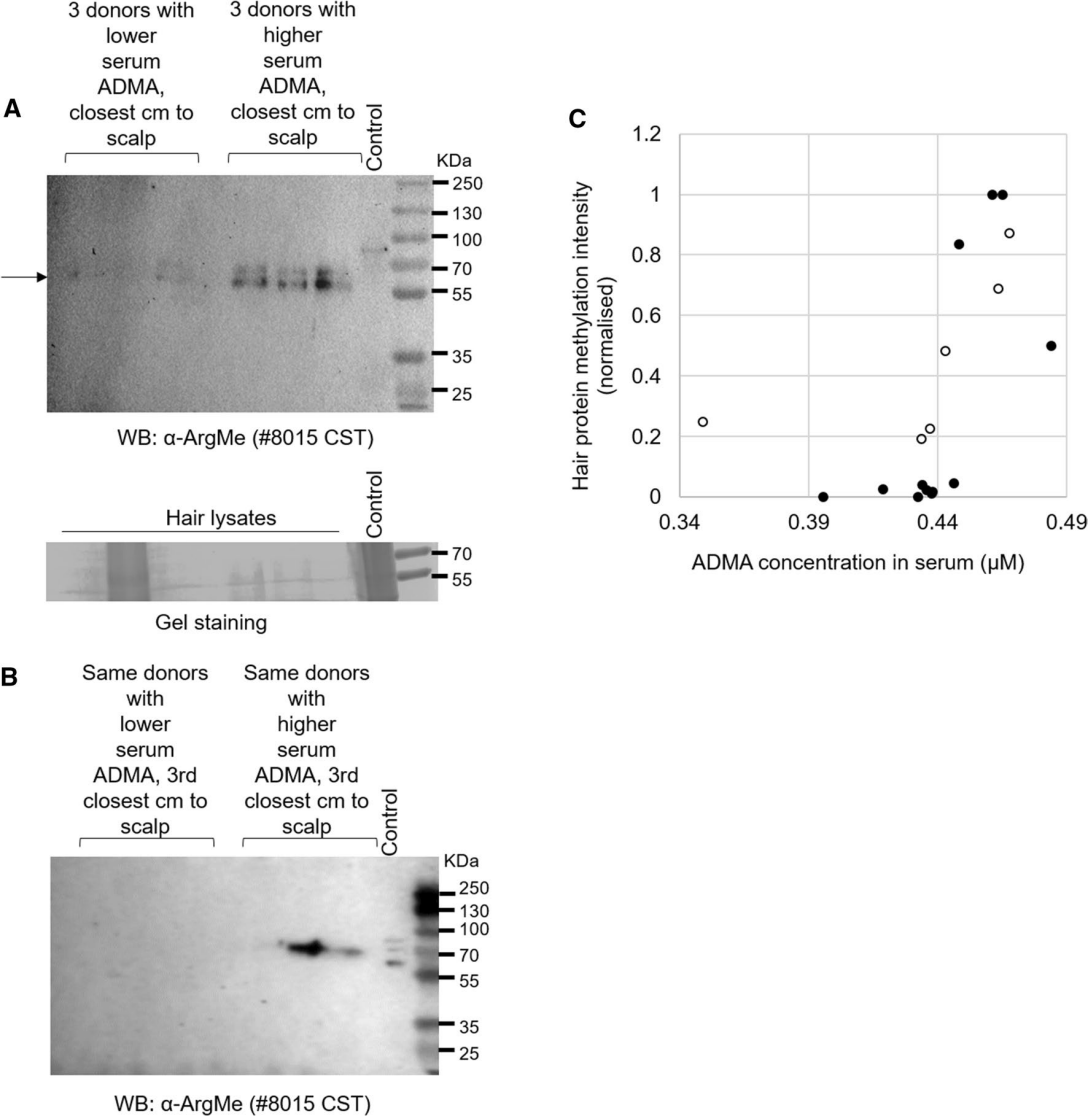
**A** Top, western blot showing increased protein ArgMe in hair proteins coming from the closest cm to the scalp of volunteers with > 0.44 µM serum ADMA levels. Bottom, same samples ran on a parallel gel, stained as loading control. The second lane from the left appears overloaded but there was little ArgMe signal on that lane. **B** Same donors as in (**A**), but in this case the 3rd closest centimetre to the scalp was analysed. **C** Plot showing a moderate positive correlation between the intensity of hair protein ArgMe and serum ADMA concentration, *n* = 18 samples. The six samples shown in panel (**A**) are identified as empty circles in panel (**C**). Pearson coefficient: 0.5302, *p* = 0.024. Spearman coefficient: 0.720, *p* = 0.00075

To provide proof of concept that levels of hair protein ArgMe may correlate with serum ADMA concentrations, we recruited 18 healthy volunteers (see Table 2 for clinical data) for collection of hair and blood samples in a small clinical study. First, we examined whether ArgMe was a stable mark on hair proteins. We ran western blots using hair protein lysates from the closest (Fig. 2A) and the 3rd closest (Fig. 2B) centimetres to the scalp of matched donors. We found that ArgMe marks were maintained on proteins at 55–70 kDa over what would correspond to a period of 2–3 months of hair growth.

**Table 2:** Clinical measurements of participants in our clinical study, sorted by ascending serum ADMA concentration. All participants in the clinical study were female

Participant No	Serum ADMA (µM)	Age (y)	Height (cm)	Weight (kg)	BP (mmHg)	Pulse (bpm)	BMI (kg/m^2^)
1	0.349	18	167	55	116/77	90	19
2	0.396	42	163	65	133/86	75	25
3	0.419	46	168	69	135/84	71	25
4	0.432	19	158	65	123/82	72	26
5	0.434	44	163	79	148/74	68	29
6	0.434	23	160	73	116/75	64	29
7	0.436	60	166	60	143/55	63	23
8	0.437	32	170	57	110/83	59	19
9	0.438	38	170	80	111/79	71	28
10	0.438	28	178	90	128/86	70	29
11	0.443	51	165	67	99/76	68	24
12	0.446	52	162	61	121/82	71	23
13	0.448	58	167	80	131/86	75	29
14	0.461	55	156	70	147/60	64	29
15	0.464	44	175	72	110/63	70	23
16	0.465	55	168	69	121/63	73	23
17	0.468	50	170	82	150/74	71	29
18	0.484	19	165	65	125/85	75	24

We then measured ArgMe of hair proteins by quantitating the intensity of the bands corresponding to methylated proteins in the closest cm to the scalp and normalised them to total protein loading. We also measured serum ADMA concentrations and we found that all values were within the expected range for healthy adults, which has long been known to be in the hundreds of nM (Fleck et al. 2003; Zakrzewicz and Eickelberg 2009), (Table 2). We observed a positive correlation between the amount of protein ArgMe in the hair and the concentration of ADMA in the serum (Fig. 2C), as indicated by a Pearson coefficient of 0.5302 and a Spearman coefficient of 0.720 (*p* < 0.05 in both cases). There were no statistically significant correlations between the levels of hair protein ArgMe (or serum ADMA concentrations) and participant age, height, weight, BP or BMI.

Taken together, our data indicate that (1) protein modification by ArgMe occurs in hair proteins and (2) levels of hair protein ArgMe correlate with those of serum ADMA in healthy volunteers.

## Discussion

Hair is an ideal surrogate tissue for biomarker discovery as it is easy to sample, has good patient compliance and few ethical issues. In this paper, we are first to report that human hair proteins are post-translationally modified by ArgMe, this is based on the recognition of hair proteins by two different antibodies specific for ArgMe, and on the analysis of mass spectrometry data. Furthermore, we propose that keratin-83 is one of the major proteins modified by ArgMe in the human hair. Within our preliminary, small cohort (*n* = 18 apparently healthy, but evidently heterogeneous donors), we show that the levels of protein ArgMe in hair correlate moderately with raised serum concentrations of a well-established cardiovascular biomarker, that is, ADMA.

Our findings complement the growing body of literature that has analysed the levels of the stress hormone cortisol in the hair as a biomarker of chronic stress, but here we make a direct link to CVD risk. The association between hair cortisol, chronic stress and CVD has been investigated and elevated hair cortisol levels have been found in patients with acute myocardial infarction (Pereg et al. 2011) and those at risk of CVD (Manenschijn et al. 2013). However, the relationship between chronic stress and CVD is undoubtedly complex (Russell et al. 2012; Wester and van Rossum 2015), with stress negatively affecting traditional risk factors for CVD including hypertension, smoking, diabetes, obesity and physical inactivity (Iob and Steptoe 2019), and chronically elevated cortisol concentrations dysregulating lipid and glucose metabolism (Geer et al. 2014; Whitworth et al. 2005).

In contrast, the biology of methylarginine metabolites is considerably simpler and a direct link may be established between high levels of circulating ADMA and the risk for CVD through the inhibition of endothelial nitric oxide synthase (Banjarnahor et al. 2020; Bollenbach et al. 2020; Craig et al. 2021; Kayacelebi et al. 2015). In the present study, we have combined the convenience of using both hair samples and methylarginine biomarkers by developing a new assay that measures ArgMe in hair proteins. Given that (1) methylarginine metabolites, including ADMA, derive from the proteolysis of proteins carrying methylated Arg residues, (2) serum ADMA concentrations have been hypothesised to correlate with average ArgMe activity across the whole body in healthy volunteers (Bollenbach et al. 2019) and renal patients (Post et al. 2021) and (3) the hair at the back of the scalp grows at approx. 1 cm per month, levels of ArgMe of proteins in each cm of hair could reflect average monthly levels of PRMT activity in hair follicles. Tissue-specific PRMT activity has been associated with metabolic disease, obesity and diabetes (vanLieshout and Ljubicic 2019). PRMT expression is increased in the retina of diabetic rats (Chen et al. 2009) and in endothelial cells exposed to low-density lipoproteins (Böger et al. 2000; Jiang et al. 2006). It is tempting to speculate that PRMT activity in hair follicles may also increase with endothelial dysfunction.

This could at least partly explain our results, which show that the levels of hair protein ArgMe in the closest cm to the scalp correlate with serum ADMA concentrations, however, the molecular basis of this correlation is likely to be more complex. Methylarginine metabolites are generated upon the degradation of proteins with methylated arginines and eliminated mainly through renal excretion either unchanged (SDMA) or metabolised (MMA and ADMA, in the form of mono- and di-methylamine, respectively), (Said et al. 2019). The enzyme responsible for the hydrolysis of MMA and ADMA into mono- and di-methylamine (and citrulline) is dimethylarginine dimethylaminohydrolase (DDAH1), (Hu et al., 2011). Consistent with this, animal studies have shown that knocking down DDAH1 increases plasma ADMA concentrations (Rodionov et al. 2019), that DDAH1 knock-out leads to CVD (Leiper et al. 2007) and that DDAH1 overexpression protects from CVD (Dayoub et al. 2008; Jacobi et al. 2010). Also, gene variants in DDAH1 have been associated with CVD in patients (Hannemann et al. 2020). Therefore, this body of evidence suggests that the levels of expression and activity of DDAH1 are key players in the development of CVD through controlling ADMA clearance (Jarzebska et al. 2019). Furthermore, the metabolism of ADMA and SDMA can also include transamination into asymmetric or symmetrical α-keto-dimethylguanidinovaleric acid, catalysed by alanine:glyoxylate aminotransferase 2 (AGXT2), and functional variants in AGXT2 may also be associated with CVD (Yoshino et al., 2014), presumably through increasing plasma ADMA concentrations.

Within this context, our work needs to be regarded as an observational study where the correlation between hair protein ArgMe and serum ADMA levels is made but we do not yet provide mechanistic insights to fully explain this correlation. Of note, the correlation between hair protein ArgMe and serum ADMA, albeit based on semi-quantitative western blot analyses, was the only statistically significant one, of those examined. Indeed, hair protein ArgMe or serum ADMA levels did not correlate with clinical parameters; as expected in a healthy cohort.

The advantage of developing a cardiovascular biomarker in hair samples is that hair biomarkers can reflect average monthly exposures retrospectively (Russell et al. 2012); characteristics not possible to achieve when single time-point blood samples or physiological measurements are collected. A related example would be the advantages offered by measuring HbA1c as a longer term measure of blood glucose concentrations across the spectrum of glucose tolerance (Mels et al. 2013). Measuring levels of protein ArgMe in hair could provide a simpler, more complete overview of chronic, but dynamic, cardiovascular risk than measurements of ADMA in serum. From this standpoint, we believe that the potentially very high value of hair protein ArgMe as novel cardiovascular biomarkers justifies and deserves further investigation.

For example, the identification of specific methylation events in hair proteins can provide a basis for dedicated diagnostics kits. Our data indicate several ArgMe sites in abundant hair proteins such as keratins and histone-associated proteins (Adav et al. 2018). We favour the hypothesis that keratin-83 is one of the major methylated proteins. This is based on (1) evidence that keratin-83 is among the most abundant proteins in the hair cuticle (Moll et al. 2008), (2) the identification of a conserved ArgMe site on keratin-83 through our proteomics data mining approach, (3) our observation that western blot signals of hair ArgMe and keratin-83 appear to overlap and (4) direct mass spectrometry analysis of keratin-83 showing at least eight ArgMe sites. The KRT83 gene encodes for a hard, type II, basic keratin-83 protein that is important in the development of hair in mammals (Liu et al. 2017), which is underscored by the fact that gene variants in KRT83 are associated with human disease (van Steensel et al. 2015). The development of antibodies specific for given keratin-83 methylation sites could accelerate future investigations of hair protein ArgMe as novel cardiovascular biomarkers, potentially using faster and more quantitative methods than western blots. Overall, our work contributes to an emerging field of research focused on protein ArgMe as biomarkers of disease and treatment, for example, recent investigations have proposed a novel cancer biomarker based on ArgMe of an RNA binding protein, hnRNP-A1, in blood mononuclear cells (Noto et al. 2020). It will be important to include cardiovascular patients and patients with typically high serum ADMA concentrations (e.g. kidney disease) in future investigations of hair protein ArgMe as biomarkers of CVD.

In conclusion, here we have provided evidence that the amounts of hair protein ArgMe correlate with the concentrations of serum ADMA, a well-known prognostic marker of CVD. This correlation is statistically significant in a small cohort of healthy volunteers, although it has to be acknowledged that we did not assess our participants for CVD risk (blood lipids, glucose, smoking, life history). The investigation of hair ArgMe through further basic research and clinical trials could deliver a technique to profile cardiovascular risk over time (including the life course) in readily available hair samples. Such samples provide a cheaper, safer, less invasive, quicker and simpler alternative to the collection of blood samples and could be collected and analysed in out-of-hospital settings. For these reasons, developing hair protein ArgMe measurements into clinically useful biomarkers could be transformational to the prevention and care of CVD, the major cause of death worldwide.

## Supplementary Information

Below is the link to the electronic supplementary material.

Supplementary file1 (DOCX 1525 KB)

Supplementary file2 (XLSX 236 KB)

Supplementary file3 (XLSX 10932 KB)

Supplementary file4 (XLSX 12 KB)
