# Supplementary material for: Love is in the hair: arginine methylation of human hair proteins as novel cardiovascular biomarkers
Source: Amino Acids. 2021 Jun 28;54(4):591–600. doi: 10.1007/s00726-021-03024-5 (PMC9117359; doi:10.1007/s00726-021-03024-5)
Supplement: Supplementary file 1 — Supplementary file1 (DOCX 1525 KB) [file 726_2021_3024_MOESM1_ESM.docx]

**Love is in the hair: arginine methylation of human hair proteins as novel cardiovascular biomarkers**

Alistair James Marsden, David RJ Riley, Stefan Birkett, Barbara-Ann Guinn, Sean Carroll, Lee Ingle, Thozhukat Sathyapalan, and Pedro Beltran-Alvarez

**Supplementary Figures 1-4.**

**
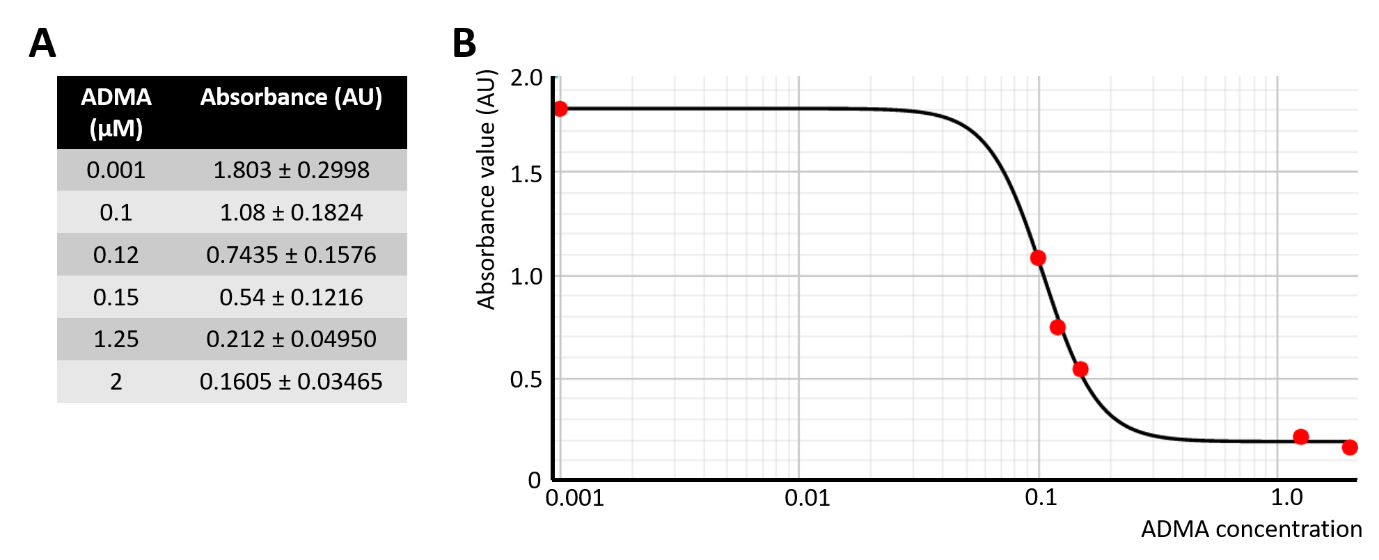
**

**Supp. Figure 1. A.** Raw data for calibration of the ELISA kit, shown as absorbance ± SD (n = 2). **B**. Calibration curve and 4-parameter logistic fit. Please note that serum samples were diluted by a factor of four before analysis, and all experimental ADMA values fell within the concentration range 0.1-0.15 µM (three middle points on the curve), with two exceptions (participants No. 1 and 2 at 0.0873 and 0.0989 µM, respectively).


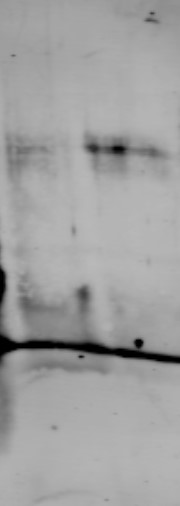


**Supp. Figure 2.** Higher exposure of lanes 7 and 8 of Figure 1B showing detection, albeit weak, of keratin-83 in these samples (arrow).


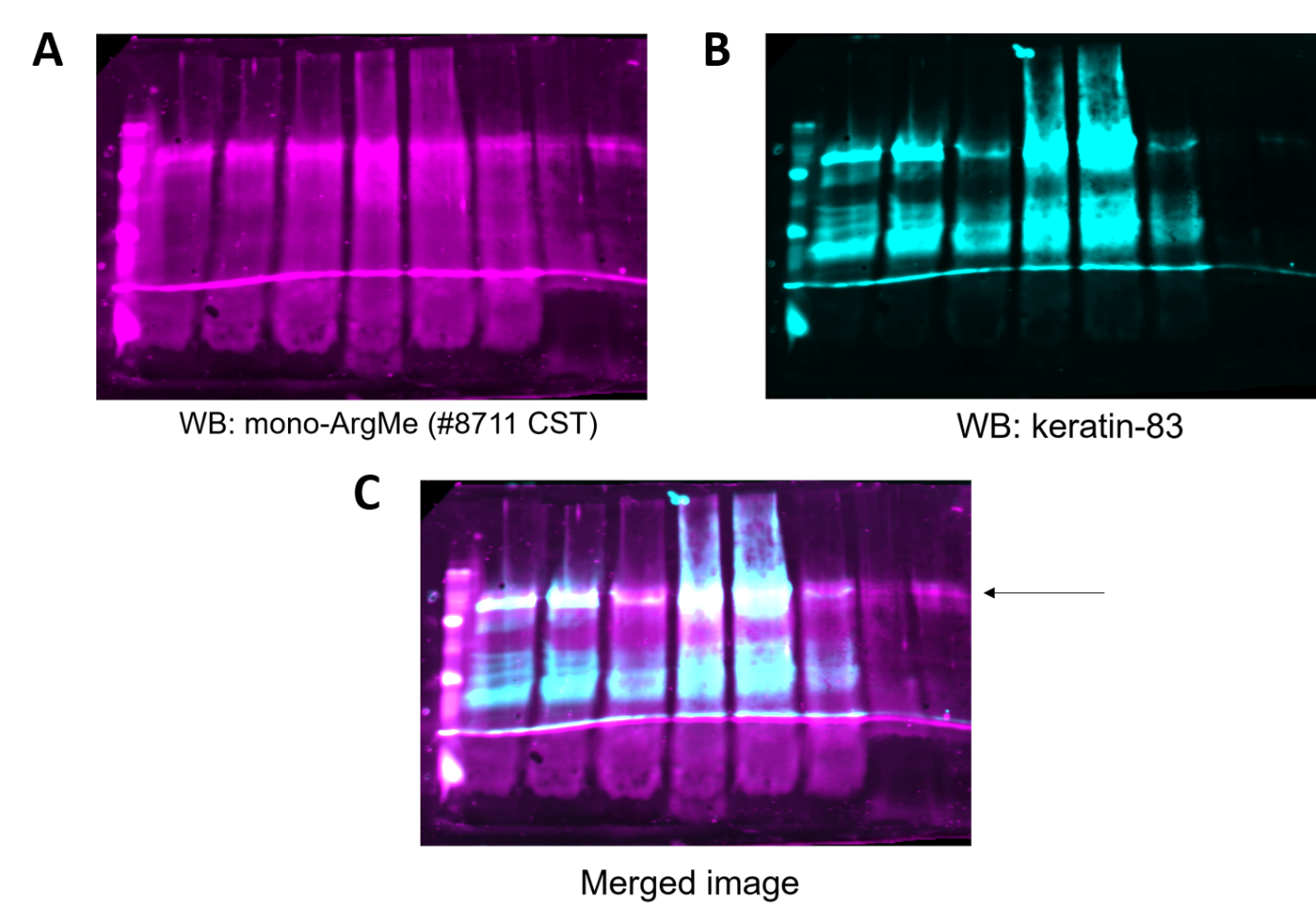


**Supp. Figure 3. A**. Same panel as in Figure 1A in the main manuscript, shown here on a pink background. **B**. Same panel as in Figure 1B in the main manuscript, shown here on a cyan background. **C**. Overlap of panels (A) and (B). The expected keratin-83 signal overlaps with the major ArgMe protein band (arrow).


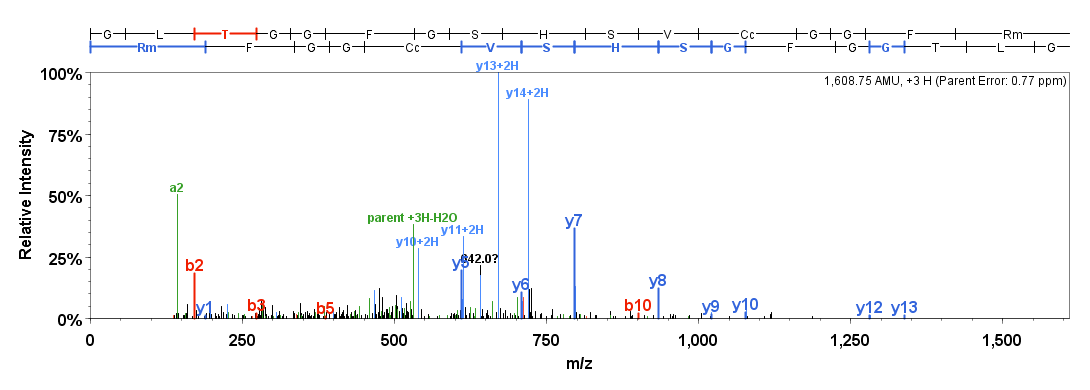


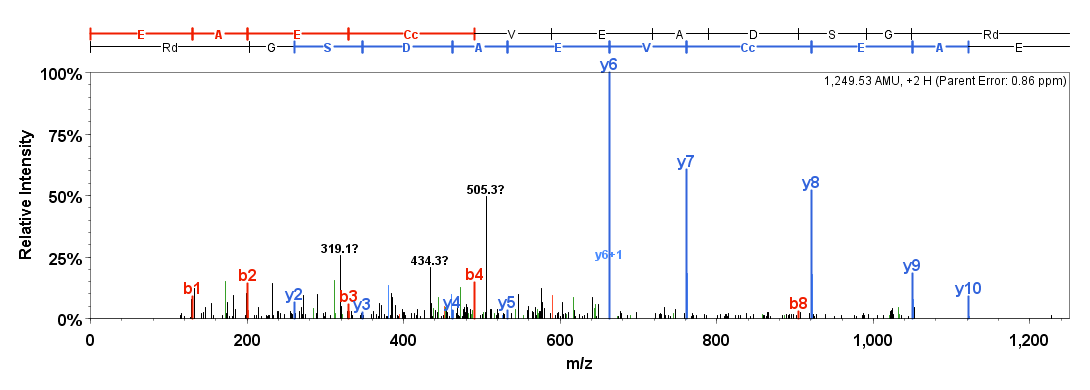


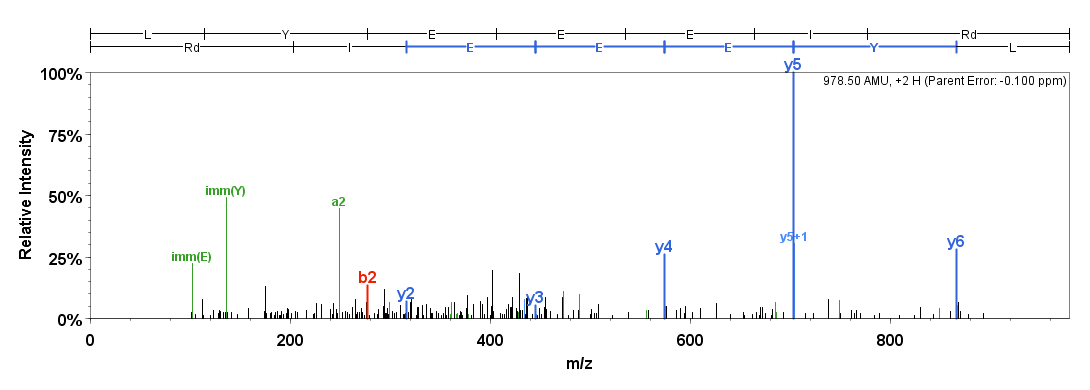


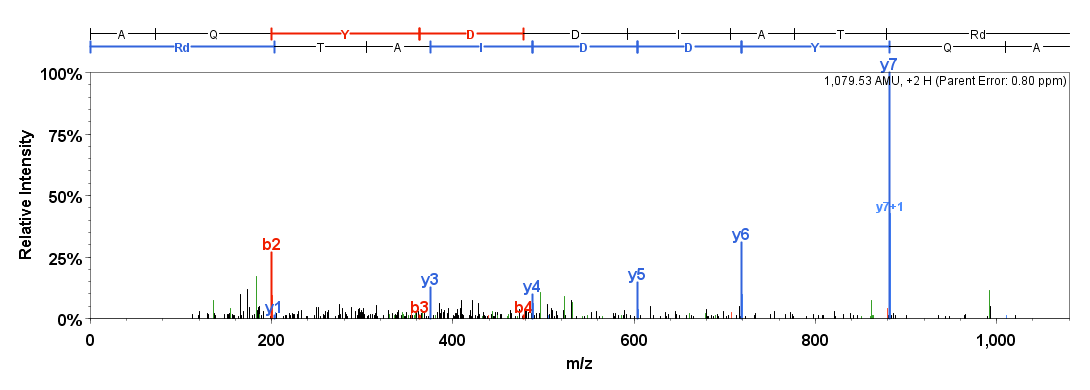


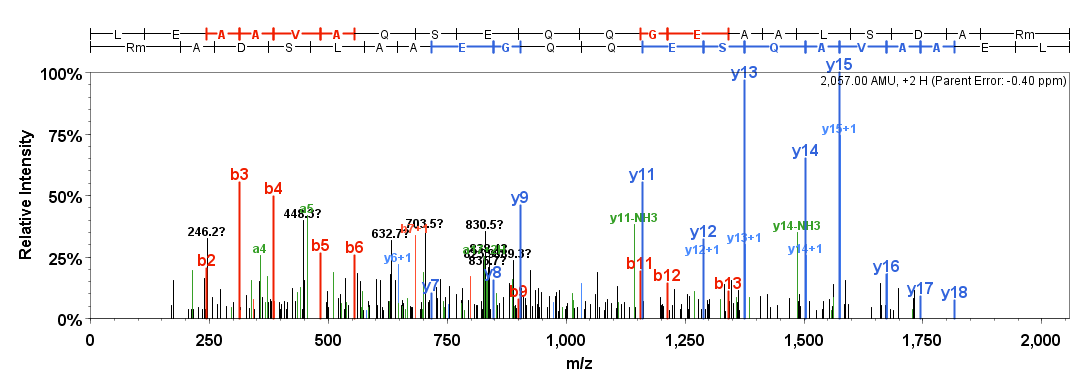


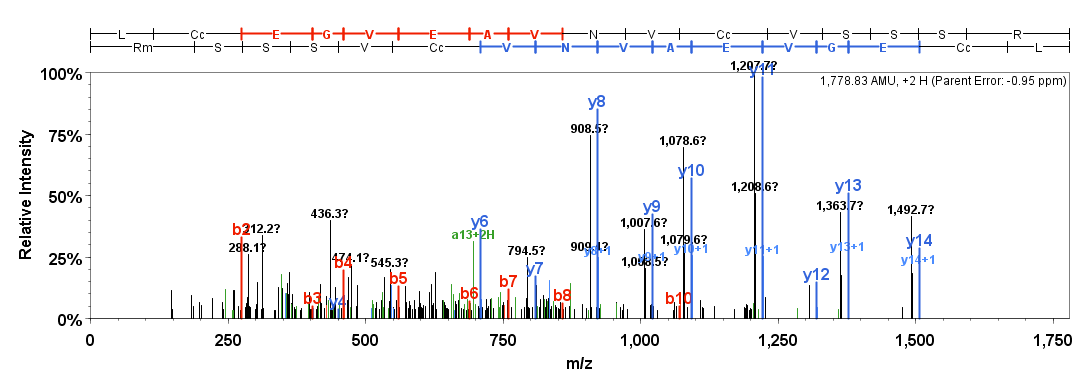


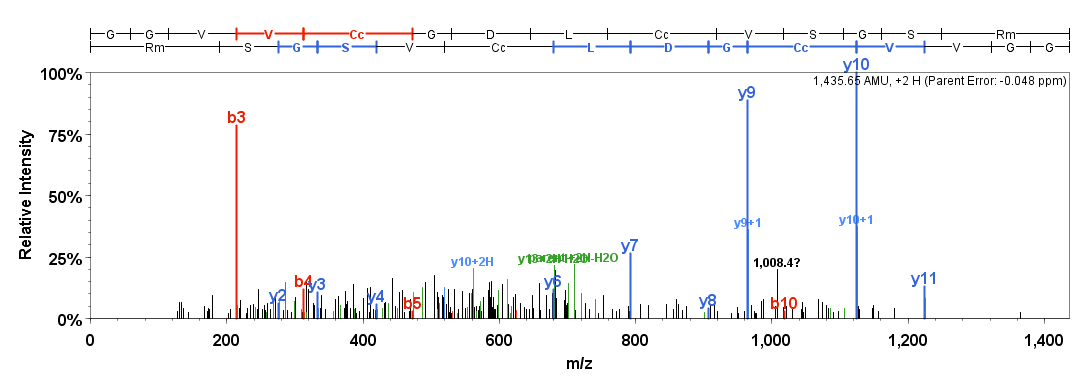


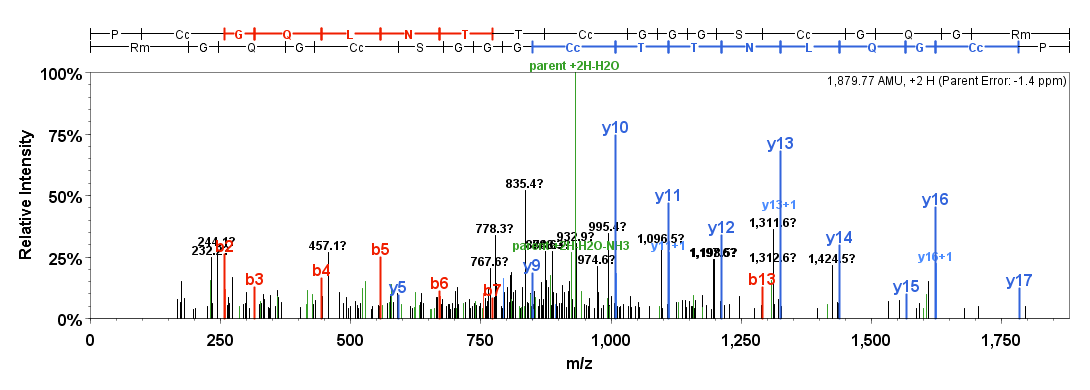


**Supp. Figure 4.** From top to bottom, individual mass spectra showing methylated R60, R180, R253, R293, R370, R427, R451 and R492. Blue and red peaks are *y* and *b* ions, respectively. Please see Supp. Table 3 for details.
